# Original Macromolecular Architectures Based on poly(ε-caprolactone) and poly(ε-thiocaprolactone) Grafted onto Chitosan Backbone

**DOI:** 10.3390/ijms19123799

**Published:** 2018-11-29

**Authors:** Cüneyt H. Ünlü, Eric Pollet, Luc Avérous

**Affiliations:** 1 BioTeam/ICPEES-ECPM, UMR CNRS 7515, Université de Strasbourg, 25 Rue Becquerel, 67087 Strasbourg CEDEX 2, France; unlucu@itu.edu.tr (C.H.Ü.); eric.pollet@unistra.fr (E.P.); 2 Science & Letters Faculty, Chemistry Department, Istanbul Technical University, Maslak, TR34469 Istanbul, Turkey

**Keywords:** chitosan, polyester, polythioester, grafting, thermal characterization

## Abstract

Polyester and/or polythioester grafted chitosan copolymers were synthesized. For that, poly(ε-caprolactone) (PCL), poly(ε-thiocaprolactone) (PTCL), and their copolymers were first synthesized by ring opening polymerization. Copolymers with caprolactone:thiocaprolactone (CL:TCL) molar ratios of 2:1, 1:1, 1:2 were synthesized. All of the synthesized macromolecular architectures were characterized using different spectral (Fourier transform infrared (FTIR), proton nuclear magnetic resonance (^1^H-NMR), X-Ray diffraction (XRD)) and thermal (Differential scanning calorimetry (DSC), Thermogravimetric analysis (TGA)) methods. Grafting was then performed according two distinct routes: (i) using a blend of both homopolymers (PCL and PTCL) or (ii) using pre-synthesized copolymers with controlled CL:TCL ratios. Hexamethylene diisocyanate was used as a grafting/coupling agent through urethane bonds with high yield. Grafting preferentially occurred at sulfur sites. The results indicated that PTCL is more reactive and favorable than PCL for grafting onto chitosan. With the homopolymers blend grafting route, the corresponding materials mostly had a higher PTCL portion than expected. To obtain polyester grafted chitosan with a determined CL:TCL ratio, the copolymer grafting route would yield better results.

## 1. Introduction

Increasing environmental concerns, the depletion of some fossil resources fractions, and the necessity to develop new macromolecular architectures have motivated the strong increase of environmentally friendly (green) alternatives for fossil-based materials during the last decade [1,2,3]. Using renewable and biobased resources as raw materials is nowadays a sustainable alternative to fossil-based polymers [1]. Being one of the most abundant naturally occurring materials on earth, polysaccharides are comprehensively largely studied due to their unique properties, including biocompatibility and biodegradability.

Chitin, which is a naturally occurring polymer that is composed of *N*-acetylglucosamine units, is the second most abundant polysaccharide on earth; it constitutes e.g., the exoskeletons of crustaceans and cell walls of some fungi [4]. Deacetylation of chitin yields chitosan, which is an aminopolysaccharide extensively studied given its antimicrobial properties and film forming capabilities [5,6,7]. Chitosan is insoluble in water, organic solvents, and aqueous bases. However, its cationic nature after amine protonation (amino groups have a pK_a_ of approximately 6.5) makes chitosan soluble in acidic solutions [8,9]. Having amine groups that are capable of forming quaternary ammonium moieties, chitosan displays antimicrobial properties. We have recently shown that chitosan modification can result in derivatives having enhanced antimicrobial activity [10]. Apart from the derivatization with ammonium salts, there are several other options to modify chitosan. One of these methods consists in grafting polymer chains onto the chitosan backbone to enhance its antimicrobial properties.

The grafting of polyester chains onto chitosan is a good way to obtain copolymers that combine the properties of both chitosan and polyesters [11]. One of the most studied aliphatic polyester is poly(ε-caprolactone) (PCL), being a biodegradable and biocompatible polymer [12]. The conventional route to synthesize PCL is ring-opening polymerization (ROP) of ε-caprolactone (CL) catalyzed by organometallic compounds, i.e., tin(II) 2-ethylhexanoate, Sn(Oct)_2_. More recently, metal-free catalysts, such as *N*,*N*-dimethylaminopyridine (DMAP) [13], 1,5,7-triazabicyclo[4.4.0]dec-5-ene (TBD) [14], and enzymes (i.e., lipases) [15,16,17,18,19,20] have gained much attention.

The substitution of a heteroatom with another one usually results in modifications on the final materials’ properties. For example, sulfur introduction to the polymer backbone of polyesters enhances thermal properties (increased melting and degradation temperatures) as well as chemical solvent resistance [15,21,22]. Thus, since polythioesters, especially poly(ε-thiocaprolactone), are potentially biocompatible, they could be used to improve or tune thermal properties of their polyesters counterparts for biomedical applications.

Although there are some studies that report the grafting of polycaprolactone onto chitosan, there is only a very limited number of studies regarding polythioesters, and, to the best of our knowledge, none of them are related to polythioester grafting onto chitosan.

The aim of this work is thus to use both polycaprolactone and polythiocaprolactone homopolymers or the corresponding copolymers for their grafting onto chitosan. The main hypothesis of this study is that grafting polyester moieties onto chitosan may synergistically enhance the final material properties. Chitosan has interesting features, such as biodegradability and antimicrobiality but limited film formation capability, which limits its use in various applications. Thus, grafting polyester moieties may enhance its film formation capability. Polycaprolactone or polythiocaprolactone grafted chitosan samples were obtained following two routes. In the first route, a blend of polycaprolactone and polythiocaprolatone was used for the grafting reaction, while the second route consisted in grafting presynthesized poly(caprolactone-co-thiocaprolactone) copolymers onto chitosan. The obtained materials were chemically and physically characterized using a large number of spectral (Fourier transform infrared (FTIR), proton nuclear magnetic resonance (^1^H-NMR), X-Ray diffraction (XRD)) and thermal (Differential scanning calorimetry (DSC), Thermogravimetric analysis (TGA)) methods.

## 2. Results and Discussions

Different preliminary and unsuccessful attempts were made to synthesize chitosan-graft-poly(ε-caprolactone) by initiating CL ROP directly from either the hydroxyl or amino groups of the chitosan backbone. Regardless, the catalyst and reaction conditions (bulk, organic/aqueous solution, metallic/organic catalyst), the obtained material had a very small yield (approximately 15%). This was probably due to strong inter/intra-chain hydrogen bonds. Possibly, these interactions permitted only first few monomers to polymerize onto chitosan, but as polyester chains started to grow longer, the chitosan backbone collapsed, preventing local diffusion of CL monomers as well as its grafting onto chitosan. Thus, another route was followed to obtain chitosan-graft-polyester. In this route the polyesters (PCL, poly(ε-thiocaprolactone) (PTCL), or copolymers) were synthesized separately then they were grafted onto chitosan.

### 2.1. Analysis of PCL and PTCL Homopolymers

**Figure 1:**
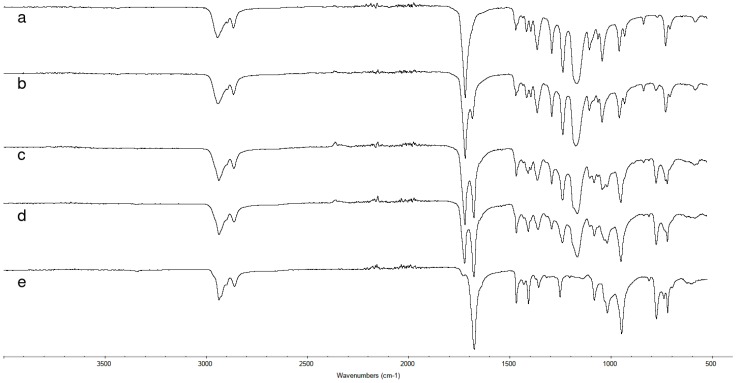
Fourier transform infrared (FTIR) spectra of (**a**) poly(ε-caprolactone) (PCL), (**e**) poly(ε-thiocaprolactone) (PTCL), and poly(ε-caprolactone-co-ε-thiocaprolactone) COP copolymers with molar proportions of (**b**) 2:1, (**c**) 1:1, and (**d**) 1:2.

**Figure 2:**
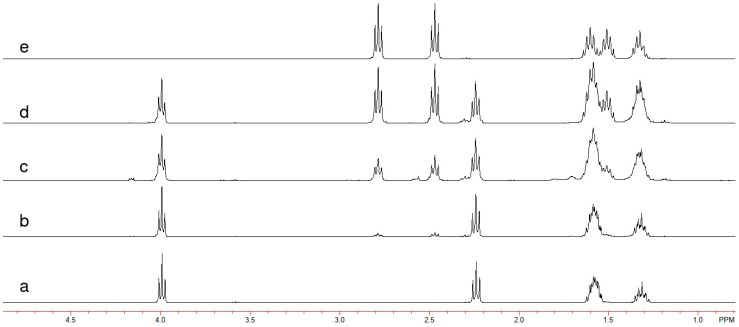
^1^H-NMR spectra of (**a**) PCL, (**e**) PTCL, and COP copolymers with molar proportions of (**b**) 2:1, (**c**) 1:1, and (**d**) 1:2.

Poly(ε-caprolactone) (PCL) and poly(ε-thiocaprolactone) (PTCL) homopolymers were successfully obtained with a yield of 97 and 75%, respectively. FTIR spectrum of PCL (Figure 1a) displayed C-H stretching vibrations with a peak position 2950 cm^−1^, a distinctive ester carbonyl, O–C=O, stretching signal at 1720 cm^−1^, and various C-O stretching and bending vibrations in 1500 and 900 cm^−1^ range, while PTCL spectrum (Figure 1e) displayed thioester carbonyl, S–C=O, stretching at 1677 cm^−1^ with other absorption signals at 1358, 1252, 949, 777, and 721 cm^−1^, which were related with the crystal structure of the thioester polymer [23]. NMR analyses also confirmed the chemical structures of PCL and PTCL. PCL was characterized by ^1^H NMR as δ = 1.23–1.40 ppm (m, 2H, –CH_2_–CH_2_–CH_2_–CH_2_CO), δ = 1.50–1.70 ppm (m, 4H, CH_2_–CH_2_–CH_2_–CO), δ = 2.24 ppm (t, 2H, –CH_2_–CO), δ = 3.99 ppm (t, 2H, CH_2_–O) (Figure 2a), and PTCL as δ = 1.25–1.41 ppm (m, 2H, –CH_2_–CH_2_–CH_2_COS), δ = 1.43–1.70 ppm (m, 4H, CH_2_–CH_2_–CH_2_–CH_2_–COS), δ = 2.47 ppm (t, 2H, –CH_2_–COS), δ = 2.78 ppm (t, 2H, COS–CH_2_–) (Figure 2e). Size exclusion chromatography (SEC) results showed that M_n_ of the PCL and PTCL were 11,000 and 6000 g/mol, with Đ of 1.7 and 2.4, respectively.

### 2.2. Synthesis and Characterization of the Copolymers

Poly(ε-caprolactone-co-ε-thiocaprolactone) copolymers were synthesized using different molar ratios of the two monomers CL:TCL in the same reaction medium. The resulting copolymers were labeled as COP, associated with the comonomers feed proportions (2:1, 1:1 or 1:2), such as e.g., “COP-2:1”.

FTIR spectra of homo- and copolymers were displayed in Figure 1b–d. From these spectra, the molar proportions of CL:TCL in the final copolymers could be calculated from the carbonyl stretching intensities. By this method, the ester content could be calculated as 73%, 54%, and 41% for COP-2:1, 1:1 and 1:2, respectively. Nearly the same results could also be obtained from integral areas of corresponding signals after deconvolution of carbonyl region (72%, 58%, and 44%). These results from the FTIR analyses were also supported by the ^1^H-NMR spectra (Figure 2b–d). The comparison of integrals corresponding to O=C–O–CH_2_ (δ = 3.99) and O=C–S–CH_2_ (δ = 2.78) indicated that 87%, 63%, and 43% ester moiety were present in copolymers COP-2:1, 1:1, and 1:2, respectively. From the FTIR spectra of the copolymers, one could also observe a broadening in carbonyl stretching vibrations that is possibly due to various (thio)ester bonds within the polymeric structure. Some shifts in positions of the peak maxima were also observed. This occurrence was attributed to the fact that the component that was higher in proportion forced the other part to a more strained conformation, causing an increase in carbonyl stretching frequency. From the SEC analyses, the M_n_ of the copolymers were determined as 4700, 3500, and 3400 g/mol with Dispersity (Đ) of 1.8, 1.7, and 1.6 for COP-2:1, 1:1, and 1:2, respectively.

These results show that, under these reaction conditions, the ε-caprolactone monomers were more eager to polymerize than ε-thiocaprolactone. A possible explanation for this behavior rests on the size and polarizability of the oxygen and sulfur atoms. Indeed, having a smaller radius and lower polarizability, oxygen forms a seven-membered ring with a higher strain and it is thus more reactive. The bulkier sulfur atom reduces the ring tension in ε-thiocaprolactone, leading to a reactivity reduction. Thus, the ROP more likely occurred on oxygen instead of sulfur-bearing cycles, resulting in a higher content of ester moiety as compared to thioester, even when thiolactone was in higher feed proportion.

**Figure 3:**
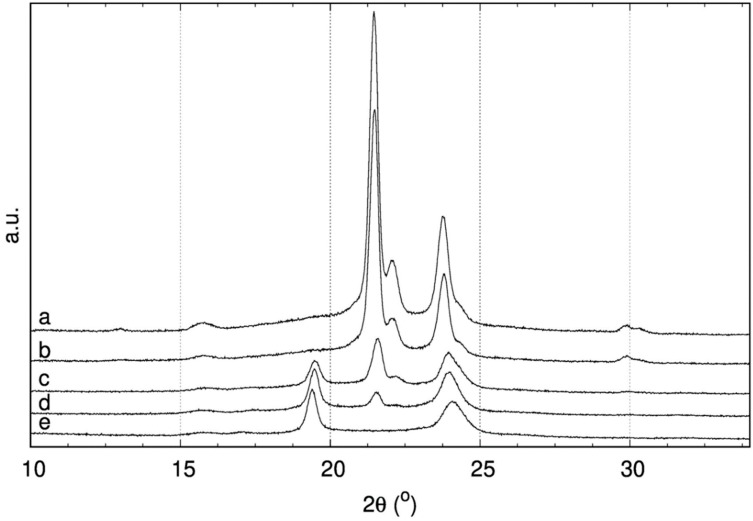
XRD diffractograms of (**a**) PCL, (**e**) PTCL, and COP copolymers with molar proportions of (**b**) 2:1, (**c**) 1:1, and (**d**) 1:2 as refraction angle (2θ, in degrees) versus count (in arbitrary units, a.u.)

XRD analyses were performed on both homo- (PCL, PTCL) and copolymers (COP-2:1, 1:1, 1:2) (Figure 3). The results of the measurements were summarized in Table 1. The XRD peaks at 2θ values of 21.5 and 23.8 degrees in the diffractograms were identified as (110) and (200) planes of pristine PCL [24]. As the TCL portion increased, the crystallite size and degree of crystallinity decreased. The crystallite size of the PCL was calculated as 4.14 Å and 3.74 Å for (110) and (200) that were planes, respectively. However, when polymerization occurred with a mixture of CL and TCL, peaks related with (110) and (200) planes shifted slightly to lower values in copolymers, resulting in an increase in crystallite size as 4.13, 4.11, and 4.12 Å for (110) plane and 3.74, 3,70, and 3.71 Å for (200) plane in COP-2:1, 1:1, 1:2 copolymers, respectively. Mean distances among chains also became smaller, supporting the decrease in crystallite size. The mean distances that were based on (110) plane were calculated as 5.18 and 5.72 Å for PCL and PTCL, respectively. Increasing TCL content caused a decrease in mean distance (5.17, 5.15, 5.16 Å for COP-2:1, 1:1, 1:2). The crystallinity degrees of the polymers were calculated based on deconvoluted peaks covering amorphous and crystalline regions. The PCL crystallinity was higher than PTCL’s one, 67% vs. 54%. The overall crystallinity of the copolymers (including both PCL and PTCL crystalline regions, where it was possible) was logically found to decrease with the PTCL content, with values of 62%, 55%, and 53% for 2:1, 1:1, and 1:2 copolymers, respectively.

**Figure 4:**
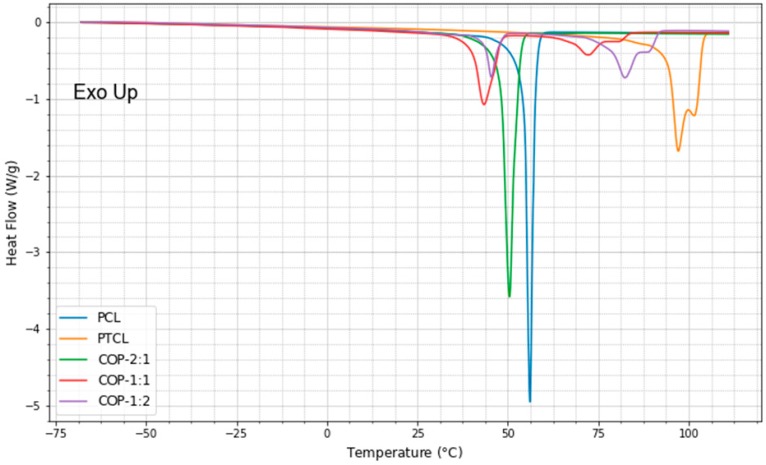
DSC thermograms of PCL, PTCL, and COP copolymers 2:1, 1:1, and 1:2 (exothermic signals shown upward).

Thermal analyses (DSC and TGA) were also conducted on homo- and copolymers. DSC results were summarized in Table 1 and Figure 4. When comparing the melting temperatures (T_m_) of homopolymers, one could observe a great difference between PCL (62 °C) and PTCL (101 °C). The exchange of the oxygen atom with sulfur induces great gain in thermal stability. However, as far as copolymers are concerned, a decreasing amount of CL resulted in a decrease in T_m_. This behavior could be due to the lower molar masses that were obtained when increasing the TCL content as well as smaller CL sequences in the copolymers.

The crystallinity of the polymers was also determined from DSC analyses. The melting enthalpies of the 100% crystalline polymer that was used to calculate the crystallinity were 139.3 J/g for PCL and 163.3 J/g for PTCL [15]. Crystallinity values of copolymers that were reported in Table 1 correspond to the overall crystallinity. The values determined from DSC analyses were consistent with XRD results. The small gap could be due to rearrangement of the polymer chains.

**Figure 5:**
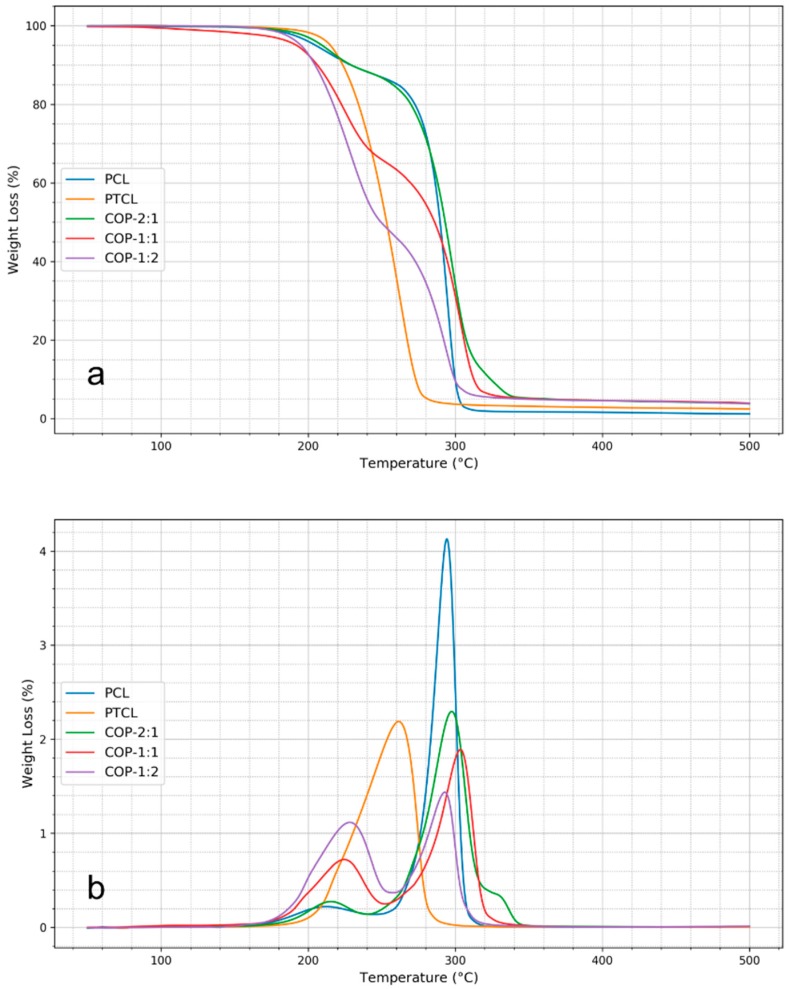
(**a**) Weight loss and (**b**) DTG curves of homo- and copolymers of CL and TCL.

TGA results were summarized in Table 2. The weight loss curves and their first order derivatives, which were recorded under argon, were shown in Figure 5. Maximum degradation temperatures (T_max_) were determined from the peak positions on derivative weight loss (DTG) plots. Thermal degradation of PCL occurred in two stages. First chain scissions (around 220 °C) took place then depolymerization reactions occurred between 250 and 320 °C [15,25,26,27]. PCL thus showed two distinct peaks in DTG plot at 212 and 294 °C, which was consistent with the results that are reported in the literature. However, PTCL showed only a broad and less intense peak centered at 262 °C, with a tailing towards 200 °C, on the DTG plot. From this result, one could say that chain scission and depolymerization occurred simultaneously. Assuming that thermal degradation reactions of the polymers were first order and they did not interfere with other reactions, the thermograms could be separated into three zones corresponding to three different thermal events (degradation reactions). Thus, the interpretations of TGA thermograms were done by splitting the measured temperature range into zones that were labelled as I, II, and III for 140–232, 232–310, and 310–400 °C intervals, respectively.

The TGA results for copolymers showed distinct variations in the first degradation stage, with T_max_ values increasing from 215 to 224, and 228 °C for COP-2:1, 1:1, and 1:2, respectively. In addition, the second stage maximum degradation temperature of the copolymers (298, 303, and 293 °C) were equal to or slightly higher than those of PCL homopolymer (294 °C). T_80%_ values were also generally higher for the copolymers with 307, 306, and 292 °C for COP-2:1, 1:1, and 1:2 copolymers, respectively, as compared to PCL (297 °C). These results tend to show that the introduction of sulfur into the polyester structure induced an overall gain in thermal stability. However, T_10%_ showed a decreasing trend with increasing content of TCL in the copolymers with 229, 206, and 205 °C for 2:1, 1:1, and 1:2 copolymers, respectively. These values were generally lower than for the PCL and PTCL homopolymers (229 and 224 °C, respectively). This indicates an earlier starting of the degradation, which is likely due to the lower molar masses of the copolymers as compared to the homopolymers. Estimated activation energy (E_A_) values were calculated, based on the Broido method, to support such gained thermal stability [28]. According to Broido, the estimation of activation energy is possible if the reactions under examination are of first order within the considered temperature range and they should not interfere with other reactions. Provided these requirements, the E_A_ could be calculated and high activation energy would correspond to high thermal stability as more energy is needed to overcome the energy barrier for degradation. As seen in Table 2, PCL shows high activation energy (109 kJ/mol) for zone I where expected reactions were mainly chain scissions. Subsequently, in zone II, there was a partial degradation reaction having an E_A_ of 36 kJ/mol. Finally, unzipping of the polymer chains mainly occurred in zone III with an E_A_ of 234 kJ/mol. PTCL displayed a different scheme with chain scissions and partial degradation occurring synchronously in zones I and II. Corresponding E_A_s were estimated as 99 and 142 kJ/mol, respectively. The last part of the PTCL degradation (zone III) required the least E_A_, which was related with main chains degradation (60 kJ/mol). These results show that, in zone I, where mainly chain scission reactions occurred, PCL and PTCL had similar behaviors, with the exception of a small difference in E_A_ of about 10 kJ/mol. However, E_A_ values for the later zones were completely different. Further analyses would depict the reaction mechanisms in detail.

Copolymers showed a hybrid behavior in between both homopolymers’ one. Indeed, COP-2:1 and 1:1 had E_A_s quite similar to PTCL in zone I. However, then they resembled to PCL with a slightly higher value for zone II, and finally display drastically different values for zone III, in between the homopolymers values. However, COP-1:2, having a higher TCL content, showed a different profile as compared to other copolymers. It displayed higher E_A_ values in first two zones, especially in zone I. Nonetheless, the E_A_ value for zone III was very close to COP-1:1. In general, and as expected, molar mass had an important effect on the E_A_. Increasing molar mass caused an increase in E_A_ as there would be more bonds to be broken in long chains (high molar mass) compared to smaller ones. Consequently, E_A_ estimations displayed that TCL amount was directly proportional with thermal stability. As chain scissions needed higher E_A_ for thio derivatives, which are more stable than oxygen-based analogues, increasing TCL portion caused an increase in thermal stability when compared to pristine PCL.

### 2.3. Grafting of Polyester Chains onto the Chitosan Backbone

FTIR spectra allowed for monitoring the reaction through the formation of carbamate bonds (1618 and 1581 cm^−1^) and the disappearance of the cyanate bond (2265 cm^−1^). After removal of the unreacted polyester chains, the remaining compound was determined as graft copolymer. First, experiments were carried out with PCL to determine the best reaction conditions, then the same conditions were used for PTCL homopolymer grafting. These chitosan graft copolymers were labelled as CG-PCL and CG-PTCL, respectively. Because of possible differences in the grafting efficiency between PCL and PTCL homopolymers chains, it might be difficult to control the PCL:PTCL ratio in the final product, which can significantly differ from the feed ratio. On the contrary, grafting copolymers could allow a better control of the CL:TCL proportion in the final material. The corresponding grafted (co)polymers were labeled according to the synthesis method as CG-B (via blending) and CG-P (via copolymers), and they were followed by the PCL:PTCL ratio.

Yields of the CG-B and CG-P samples are given in Table 3. In the case of homopolymers, the yield was higher for CG-PTCL when compared to CG-PCL. According to these results, one could say that PTCL grafting was slightly easier than PCL chains grafting onto chitosan. When both PCL and PTCL were added as a blend and the proportion was in favor of PCL (CG-B-2:1), the grafting yield (54%) was lower as compared to homopolymers’ grafting (CG-PCL 70%, CG-PTCL 81%). An increasing proportion of PTCL in blend caused an increase in yield, but it remained lower when compared to homopolymer analogues. As far as blend grafting samples are concerned, the low PCL content also confirmed the preferential (easier) grafting of PTCL onto chitosan.

Results on grafted copolymers were consistent with homopolymer blends ones. The yields and grafting efficiencies were similar to those of the equivalent homopolymer that was present in higher proportion. For example, CG-P-2:1, displayed a yield value of 71%, which was very close to CG-PCL (70%). Similarly, CG-P-1:2 showed a 76% yield, while CG-PTCL had 81%. These results indicated that coupling via sulfur atom was more favorable when compared to oxygen analogue as a consequence of the activities difference between -SH and -OH ending groups on the polymer chains.

**Figure 6:**
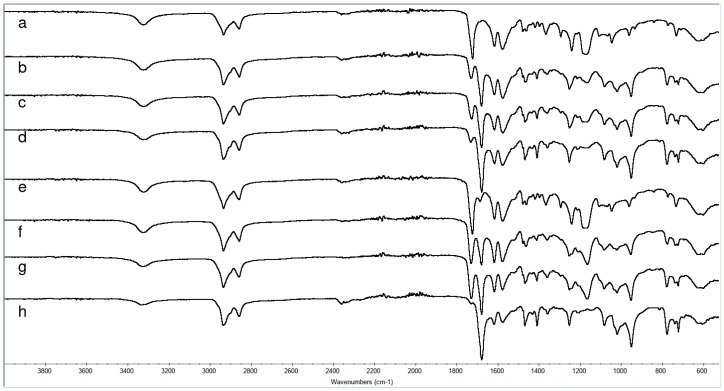
FTIR spectra of chitosan graft copolymers; (**a**) CG-PCL, (**b**) CG-B-2:1, (**c**) CG-B-1:1, (**d**) CG-B-1:2, (**e**) CG-P-2:1, (**f**) CG-P-1:1, and (**g**) CG-P-1:2, (**h**) CG-PTCL.

FTIR spectra of chitosan and chitosan grafted with copolymers (CG) were recorded (Figure 6).

Characteristic FTIR signals that were related to chitosan were summarized as O-H and N-H stretching vibrations (overlapped) as a broad signal with peak positions at 3357, and 3282 cm^−1^, various C-H stretching with a peak at 2870 cm^−1^, amide I and/or adsorbed water vibration at 1648 cm^−1^, amide II vibration at 1589 cm^−1^, various C-C-H, C-O-C, C-C-O bending, and C-C, C-O stretching vibrations between 1500–1800 cm^−1^, and anomeric (C_1_) related signal at 897 cm^−1^ indicated β- conformation of the chitosan. Chitosan grafted with copolymers or homopolymers blends displayed the characteristic urethane functional group signals as N-H stretching (3324 cm^−1^), N–C(=O)–O stretching bands 1615 and 1576 cm^−1^. In addition, the disappearance of the N=C=O stretching signal at 2266 cm^−1^ confirmed the formation of urethane bonds.

PCL content of the grafted chitosan materials (for both CG-B and CG-P) were estimated using intensity values of ester and thioester carbonyl stretching vibrations, as for COP samples. For CG-B samples, the PCL content was measured around 28% (by weight) for 2:1 and 1:1 blends. However, it dropped to 15% for 2:1 blend composition. For COP-2:1, 1:1, and 1:2 copolymers, the estimated PCL content was 74%, 49%, and 39% for CG-P-2:1, 1:1, and 1:2, respectively. These results brought another support for the preferential grafting of PTCL over PCL.

**Figure 7:**
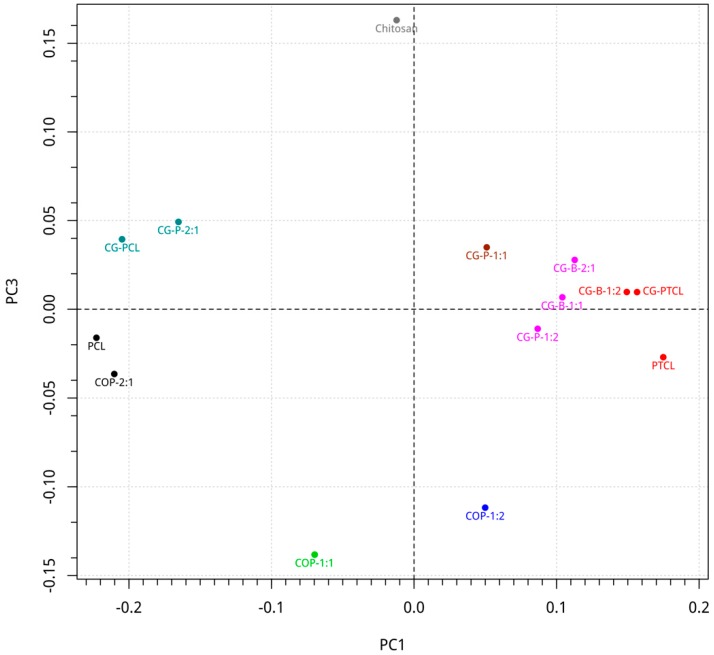
Scores plot of principal component analysis (PCA) applied to FTIR data.

The C-O groups’ stretching vibrations of chitosan and polyester samples overlapped in the fingerprint region. Moreover, higher intensity of ester group vibrations completely covered the signals of chitosan and prevented the direct observation and confirmation of grafting onto chitosan backbone. Similarly, urethane bond vibrations also overlapped with amide I and amide II vibrations. In order to detect and classify the differences in these vibration signals, a statistical method, PCA, was applied to the FTIR data. The scores plot of the first three principal components (PC), which together account for 93% of total variance, is shown in Figure 7. The plot displayed PC1 and PC3 as axes, colors as PC2, with same colors indicating close groups in three-dimensional (3D) space. Output of the PCA gave PCL, PTCL, and chitosan at different positions of the PC1–PC3 plane; PCL located at the far left and PTCL at the far right of PC1 axis, while chitosan was in the middle. However, both PCL and PTCL were fairly below the zero level of PC3, while chitosan was clearly away from them. These points created a triangular shape whose corners were occupied by one component making them clearly distinguishable. In this shape, COP copolymers lined up at the farthest distance from chitosan based on their composition. Chitosan graft copolymers also showed a similar lining up but they were closer to chitosan region. CG-PCL and CG-P-2:1 showed up at lower left part of the triangle, close to PCL position, but slightly shifted upper part towards chitosan; then, CG-P-1:1 were near to the origin of the plot. The other chitosan grafted copolymers and CG-PTCL were very close to the PTCL homopolymer, but also shifted to the chitosan side of the PC3 axis. These results were in accordance with other results, as all CG-B copolymers and CG-P-1:2 copolymer had a higher proportion of thioester component in their compositions. This result also indicated that functional groups were all different in each copolymer and attested that these samples are composed of various covalent bonds between chitosan and (co)polymers instead of a physical mixture. Thus, it could be concluded copolymers chains were effectively grafted onto the chitosan backbone.

XRD diffractograms were recorded and the main results were summarized on Table 1. In the 2θ angle region corresponding to the (110) plane, grafted copolymers displayed two distinct peaks corresponding, respectively, to PCL and PTCL homopolymers. The presence of these two peaks likely attested that CG copolymers had distinct partially crystalline poly(thio)ester blocks. However, the (200) plane peaks were overlapped, so it was not possible to separate distinct signals. Thus, interchain distances were calculated based on the (200) plane peaks using Equation (4). In general, the interchain distance was nearly constant for chitosan grafted copolymers as compared to their parent samples. For example, PCL, COP-2:1, CG-PCL, and CG-P-2:1 displayed an average interchain distance of 4.67 Å. The introduction of thio- component caused a small decrease in this value by 0.04 Å, with an average value of 4.63 Å. The absence of crystalline peaks on the diffractogram for neat chitosan confirmed that it was an amorphous material. As expected, grafting the copolymers onto chitosan caused a decrease in their crystalline structure. Crystallinity of the samples were calculated using Equation (5); the average crystallinity value was found to be around 30% for chitosan graft samples, while it was around 50% for the corresponding non-grafted homo- and copolymers.

Thermal characterizations of homo- and copolymers, as well as chitosan-grafted copolymers, were carried out by DSC (Table 4). The thermograms of chitosan grafted with homopolymers of PCL and PTCL (CG-PCL, CG-PTCL) showed T_m_ values similar to the corresponding pristine homopolymers. However, there was a sharp decrease in crystallinity for the grafted samples. The materials with varying CL:TCL ratios displayed two melting zones each corresponding to PCL and PTCL melting zones. One could also observe that CG-B copolymers thermograms resembled the PTCL one with only small differences. This could be explained by the FTIR results that showed that CG-B copolymers were rich in the PTCL component. Some discrepancies on crystallinity were observed between the χ_DSC_ and χ_XRD_ results. According to XRD, the crystallinity values of chitosan grafted with homopolymers blends were estimated around 35%, whereas from DSC the estimated crystallinity of these CG-B samples were calculated as 10%, 46%, and 57% for 2:1, 1:1, and 1:2, respectively. Similarly, lower values were estimated for the CG-P series as 20, 11, 30% for 2:1, 1:1, and 1:2. However, it is very well known that DSC and XRD often give divergent crystallinity values for a same sample.

Thermogravimetric analysis of chitosan displayed 10% weight loss below 100 °C due to water loss. Then, thermal degradation started at 220 °C giving a T_max_ at 274 °C. After 380 °C, the degradation rate was constant until the end of analysis. TGA results were summarized in Table 5. An additional thermal decomposition zone (IV) was observed between 400 and 460 °C for grafted samples giving an average T_max_ value of 410 °C.

When compared to PCL and PTCL homo- and copolymers, chitosan grafted samples generally displayed higher thermal stability with an additional T_max_ just above 400 °C, probably related to the chitosan degradation. The polyester chains grafting onto chitosan led to increased temperatures in the first part of thermal degradation, which was related to PCL or PTCL chain scissions, as their coupling to chitosan via urethane bonds likely prevented this reaction. Then, chain unzipping reactions also peaked at higher temperatures as compared to pristine (co)polymers. The temperatures at which 10% weight loss occurred also supported this gain in thermal stability. The initial and final positions of degradations were similar in CG-B and CG-P graft copolymers. However, they differed slightly in CG-PCL and CG-PTCL. The E_A_ for CG-PCL was calculated according to the initial and final points at 180 and 460 °C with two break points around 235 and 322 °C. The last two reaction zones were likely related to urethane bond decomposition and chitosan degradation as evidenced by the similar E_A_ values obtained for all CG-samples regardless their types and composition. Thus, only the first two degradation zones showed differences in the thermal stability of the samples according to the CL:TCL ratio. For CG-B samples, E_A_ values for zone I were 30% and 5% higher when compared to CG-PCL and CG-PTCL, respectively. However, E_A_ values for zone II, which was related to PCL moiety degradation, were nearly 50% lower than for CG-PCL. However, CG-P samples displayed a slightly different scheme with E_A_ values related to the TCL content. In zone I, approximate increases of 30%, 50%, and 60% were observed as the TCL content increased, while E_A_ values in zone II showed a decrease by 20%, 50%, and 60%. These results indicated that the introduction of sulfur atoms into the polyester chains structure induced a gain in thermal stability for the chitosan grafted copolymers as well.

### 2.4. Film Formation of the Materials

**Figure 8:**
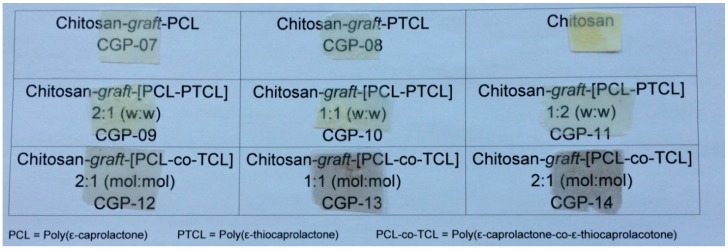
Films obtained from chitosan and chitosan-grafted (co)polymers.

Thin films were prepared by hot pressing for all graft samples (CG-PCL, CG-PTCL, CG-B, and CG-P polymers) and neat chitosan. The resulting films were shown in Figure 8. As neat chitosan had adsorbed water in its structure, a film formation under hot pressing conditions was possible, but the obtained film was very opaque and brittle. Other film samples were quite transparent and resistant when compared to chitosan film.

## 3. Experimental

### 3.1. Chemicals

Chitosan (ChitoClear^®^, degree of deacetylation (DD) = 97% and mass-average molar mass (M_w_) = 250–300 kDa) was purchased from Primex (Siglufjordur, Iceland) as a white powder with particle diameter of less than 1 mm (100% through mesh 18) and was used as received; ε-caprolactone (99% for synthesis) was purchased from Sigma Aldrich (Saint-Quentin Fallavier, France) and distilled over CaH_2_ prior each use. Tin(II) 2-ethyl hexanoate (Sn(Oct)_2_) and 1,5,7-triazabicyclo[4.4.0]dec-5-ene (TBD) were purchased from Sigma Aldrich (Saint-Quentin Fallavier, France) hexamethylenediisocyanate (HDI) from Fluka (Seelze, Germany), they were used as received without further purification. Anhydrous toluene was freshly distilled over sodium under argon atmosphere. Other solvents (GC grade) were purchased from Acros Organics (Illkirch, France) and were used without further purification.

### 3.2. Methods:

FTIR spectra were recorded on a Thermo Nicolet iS10 equipped with an ATR sampling module with a diamond crystal. Measurements were done with 32 scans with a spectral resolution of 4 cm^−1^.

Principal component analysis (PCA) was used for data reduction in order to find out the dominant factors. A typical FTIR spectrum was composed of several hundreds of points with a correlation to each other. Using PCA, this correlation was removed in a way creating uncorrelated variates known as principal component (PC) scores. The PCs could be defined as new, orthogonal axes where variances were maximized. Thus, the projection of PCs against one another could reveal the clustering or structural information regarding the major components that are responsible for the change in certain region of the spectrum. PCA was performed on first derivative FTIR data (including the homo- and copolymers) in order to show the evidence of the grafting onto chitosan using R software (version 3.4.3) running on a 64-bit Linux platform (kernel version 4.14.15). First-order derivatives of the spectra in range between 1800 and 700 cm^−1^, which were calculated via the Savitzky-Golay algorithm averaging four-points left and right side using a second order polynomial, were used in order to eliminate the errors that could arise from overlapping.

^1^H-NMR spectra were measured on a Bruker instrument at 400 MHz (^1^H) at 25 °C using deuterated solvents (32 scans).

Size exclusion chromatography (SEC) was performed to determine the number-average molar mass (M_n_), M_w_, and the dispersity (Ð) of the samples. Measurements were performed in chloroform (HPLC grade) in a Shimadzu liquid chromatograph that was equipped with a LC-10AD isocratic pump, a DGU-14A degasser, a SIL-10AD automated injector, a CTO-10A thermostated oven with a 5 μ PL-gel Guard column, two PL-gel 5 μ MIXED-C and a 5 μ 100 Å 300 mm columns, and two online detectors, a Shimadzu RID-10A refractive index detector, and a Shimadzu SPD-M10A diode array (UV) detector, respectively. Molar masses and dispersity were calculated from a calibration with polystyrene standards (580 to 1,650,000 g/mol).

Thermogravimetric analysis (TGA) measurements were conducted under argon (at a flow rate of 25 mL/min) using a Hi-Res TGA Q5000 apparatus from TA Instruments. The samples (2–5 mg placed in a platinum pan) were heated up to 500 °C at 10 °C/min. Activation energy was obtained from the plot of Equation (1), where *E_A_*, *R*, and *T* were defined as activation energy (kJ/mol), universal gas constant (8.314 J mol^−1^ K^−1^), absolute temperature (K); *y* was the fraction, which was defined as Equation (2) where *Wt*, *W_∞_*, and *W*_0_ were the sample weight at temperature *t*, the end and beginning of decomposition, respectively [28].

(1) $$\ln\left(\ln\left[\frac{1}{y}\right]\right)=\frac{E_{A}}{R}\frac{1}{T}$$

(2) $$y=\frac{W_{t}-W_{\infty }}{W_{0}-W_{\infty }}$$

Differential scanning calorimetry (DSC) measurements were performed using TA instruments DSC Q200 apparatus. The measurement method had three cycles; first, the sample was heated up to 120 °C at a heating rate of 20 °C/min and was kept at this temperature for 1 min to remove adsorbed water and to erase the thermal history. Subsequently, it was cooled down to −70 °C with a cooling rate of 5 °C/min and was kept at this temperature for 1 min. Finally, the sample was heated up to 150 °C with a ramp of 10 °C/min. All cycles were conducted under nitrogen flow (50 mL/min). Crystallinity (*X_DSC_*) was calculated according to the Equation (3), where Δ*H_m_* and $\Delta H_{m}^{0}$ are the melting heat of the measured sample and of a 100% crystalline polymer, respectively. The melting heat of the sample was measured on the last cycle.

(3) $$\chi_{DSC}=\frac{\Delta H_{m}}{\Delta H_{m}^{0}}\times 100$$

X-ray diffraction (XRD) patterns were recorded with a Bruker AXS D8 ADVANCE using Cu-K_α_ radiation (*λ* = 0.1542 nm) operating at 40 kV and 40 mA. The scattering range was 2θ = 10–50°, with a step size of 0.0158° and a scan speed of 0.5 s/step. The mean distance among the closest neighboring chains (*R*) was determined with Equation (4) [29].

(4) $$R=\frac{5}{8}\left(\frac{\lambda }{\sin\theta }\right)$$

Crystallinity (*X_XRD_*) was calculated using Equation (5), where *I_cry_* and *I_total_* were the area under the curve of crystalline peaks and total integral of all peaks. Crystalline and amorphous peaks were deconvoluted using Fityk software (version 1.3.1).

(5) $$\chi_{XRD}=\frac{I_{cry}}{I_{total}}\times 100$$

Films of the materials were prepared by compression molding using a Labtech LP-S-20 hot press (Labtech Engineering Company, Muang, Thailand). Polymers samples (150 mg) were placed between two steel plates that were covered with Teflon layers and pressed at 90 bar and 110 °C for 2 min. After cooling (5 min), the resulting films were removed and photographed.

### 3.3. Materials Synthesis

#### 3.3.1. Synthesis of 6-Mercaptohexanoic Acid

**Scheme 1:**
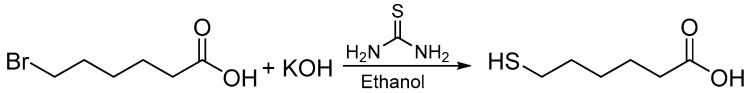
Synthesis of 6-mercaptohexanoic acid.

6-mercaptohexanoic acid synthesis protocol was adapted from Shimokawa et al. [30]. The reaction is shown in Scheme 1. Briefly, 6-bromohexanoic acid (70 mmol, 13.66 g) was dissolved in ethanol (9.1 mL) and added dropwise to hot (80–90 °C) solution of thiourea (70 mmol, 5.33 g) in water (46 mL) under continuous stirring. The mixture was heated to reflux (90–100 °C) for 2 h. Following, KOH (10 g) in 30 mL water was added and the reaction was allowed to proceed at 90–100 °C for one day. Medium was cooled down to room temperature and then acidified with HCl solution (1 mol/L) until neutral pH was reached. Mixture was extracted with chloroform and washed three times with water. Finally, solvent was stripped off by rotary evaporation at 35 °C under reduced pressure (40 mbar). A typical yield of 90% was obtained.

FTIR of the product displayed C=O stretching at 1704 cm^−1^, acidic O-H stretching as a broad signal between 3500–2400 cm^−1^, S-H stretching at 2666 cm^−1^ as a weak band.

The final product was characterized by NMR spectroscopy: ^1^H NMR (400 MHz, CDCl_3_): δ = 1.35 ppm (t, 1H, HS–), δ = 1.40–1.50 ppm (m, 2H, –CH_2_–CH_2_CH_2_CO_2_H), δ = 1.57–1.70 ppm (m, 4H, –CH_2_–CH_2_–CH_2_–CH_2_CO_2_H), δ = 2.36 ppm (t, 2H, –CH_2_–CO_2_H), δ = 2.52 ppm (m, 2H, HS–CH_2_–).

#### 3.3.2. Synthesis of ε-Thiocaprolactone (TCL)

**Scheme 2:**
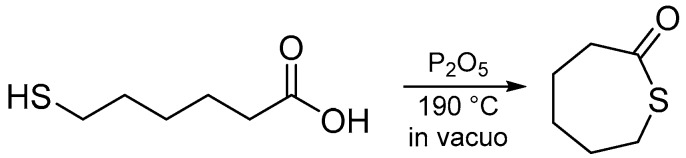
Synthesis of ε-thiocaprolactone.

The ε-thiocaprolactone synthesis protocol was adapted from Overberger et al. [23]. Typically, 6-mercaptohexanoic acid (27 mmol, 4 g) was placed in a 50 mL round bottom flask. 0.8 g of P_2_O_5_ was added. The flask was quickly fitted with a short distillation head. Subsequently, the flask was immersed in an oil bath at 190 °C and the pressure was lowered to 80 mbar. The distillation rate of thiolactone was maintained by lowering pressure gradually until 1 mbar. After 2 h, thiolactone was solubilized in ether and was washed with aqueous NaHCO_3_ until it reached neutral pH. Mixture was then dried over magnesium sulfate and ether was stripped off by rotary evaporation, typically giving a yield of 40% (Scheme 2).

FTIR spectrum confirmed the ring-closure with the disappearance of the acid O-H broad stretching signal between 3500–2400 cm^−1^ and the shift of the C=O stretching to 1656 cm^−1^.

The final product was characterized by 1H NMR (400 MHz, CDCl_3_): δ = 1.70–1.90 ppm (m, 4H, –CH_2_–CH_2_–CH_2_–CH_2_–COS), δ = 2.05–2.15 ppm (m, 2H, COS–CH_2_–CH_2_), δ = 2.85 ppm (t, 2H, –CH_2_–COS), δ = 3.05 ppm (t, 2H, COS–CH_2_–).

#### 3.3.3. Synthesis of poly(ε-caprolactone) (PCL) and poly(ε-thiocaprolactone) (PTCL) Homopolymers

**Scheme 3:**
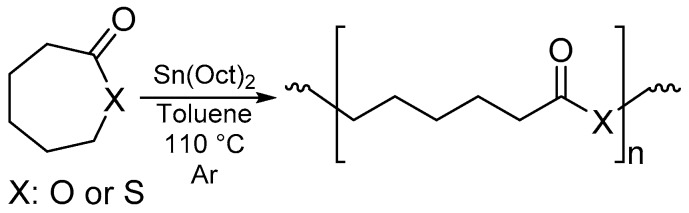
Synthesis of homo- and copolymers from ε-caprolactone and ε-thiocaprolactone.

ε-caprolactone (CL) (2.1 mL, 18.86 mmol) was dissolved in dry toluene (10 mL) under argon atmosphere and was heated to 110 °C. Afterwards, 1.25 mL of Sn(Oct)_2_ solution (5% in toluene) was added to the mixture. After 24 h the reaction was stopped by addition of one drop of 1 M HCl solution to the reaction mixture. Polymer was precipitated by addition of heptane, then filtrated and dried first in air then in vacuum for 24 h (yield 97% by weight) (Scheme 3).

The same procedure was applied with TCL (2.4 mL, 18.64 mmol) to obtain poly(ε-thiocaprolactone) (PTCL) (yield 75% by weight).

#### 3.3.4. Synthesis of poly(ε-caprolactone-co-ε-thiocaprolactone) Copolymers

Poly(ε-caprolactone-co-ε-thiocaprolactone) copolymers were synthesized using the above procedure with different ε-caprolactone/ε-thiocaprolactone feed ratios (mol/mol). The use of 2:1 ratio resulted in a yield of 64% by weight and the obtained product was labeled as COP-2:1. Same protocol was repeated using comonomers molar proportions of 1:1 and 1:2. Final product yields were 40 and 32% by weight for COP-1:1 and COP-1:2, respectively.

#### 3.3.5. Grafting Polyesters onto Chitosan

**Scheme 4:**
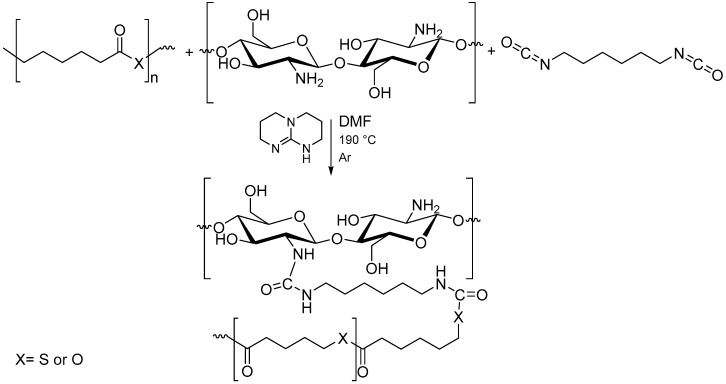
Reaction of polyester chains grafting onto the chitosan backbone.

In a typical procedure polyester (300 mg), TBD (1.6 mg, 0.01 mmol), and chitosan (100 mg, 0.6 mmol NH_2_ equivalent) were stirred in DMF (3.2 mL) at 100 °C, under argon. After 20 min, HDI (0.13 mL, 0.8 mmol) was added to the mixture and was left under stirring for 90 min. The mixture was then poured into ice water to precipitate copolymer. The obtained chitosan-graft-polyester copolymer was washed with acetone to remove unreacted polyester. Finally, it was extracted with toluene using Soxhlet apparatus for 24 h and dried first in air then in vacuum. The overall procedure was given in Scheme 4.

The grafted copolymer yield was calculated using Equation (6).

(6) $$Yield,\;\%=\frac{W_{graft}}{W_{polymer}+W_{chitosan}}\times 100$$

where *W_graft_* weight of the polymer-graft-chitosan, *W_polymer_* was the weight of polyester, polythioester, or copolymer, and *W_chitosan_* was the weight of chitosan.

## 4. Conclusions

Homopolymers and copolymers of CL and TCL were successfully synthesized. Copolymerization that was performed with different monomers molar ratios yielded copolymers in favor of PCL resulting from the easier polymerizability of CL. Although the molar masses of the PTCL and COP were smaller, they showed better thermal stability when compared to PCL. The crystallinity degree of COP samples was found lower when compared to PCL and PTCL homopolymers. The obtained homo- and copolymers were then coupled to chitosan backbone using isocyanate to obtain chitosan grafted copolymers (CG-), according two distinct routes. In the blending route, homopolymers mixtures were used, while in the second route, presynthesized COP with varying ratios were grafted. The latter route was found to be superior as the PCL:PTCL ratio could be adjusted as desired. Obtained CG- samples had even better thermal stability, but lower crystallinity when compared to corresponding polyesters. Grafting onto chitosan via isocyanate, groups preferentially occurred at sulfur sites. Thus, the homopolymer blends grafting route yielded grafted polyesters in favor of PTCL. The hot-melt pressing of grafted copolymers yielded flexible and durable films.

To answer the initial hypothesis and objective, grafting polyester chains onto chitosan enhanced the properties of the starting materials, as expected. In comparison to pristine starting materials, grafted copolymers had better thermal stability and film forming capabilities. The PCL/PTCL proportion could be tuned via copolymerization prior to grafting. Then, using presynthesized copolymers for grafting would yield well defined and controlled PCL/PTCL ratios in the final material.

Complementary studies should be performed to analyze in details some functional properties, such as the mechanical, drug release, and antimicrobial behaviors of CG- copolymer films in connection with specific biomedical applications, such as e.g., active wound dressing.

## Figures and Tables

**Table 1:** Main results from X-Ray diffraction (XRD) and Differential scanning calorimetry (DSC) analyses of homo- and copolymers of ε-caprolactone (CL) and ε-Thiocaprolactone (TCL).

Sample	2θ (110)		2θ (200)	Chain Distance ^a^	Χ_XRD_^b^	T_m, I_^c^	T_m, II_^c^	CL ^d^	ΔH_m_	Χ_DSC_^e^
	(°)		(°)	(Å)	(%)	(°C)	(°C)	(%, mol/mol)	(J/g)	(%)
PCL		21.5	23.8	4.68	67	56	-	100	111.8	80
PTCL	19.4		24.1	4.61	54	-	97	0	71.9	44
COP-2:1		21.5	23.8	4.67	62	50	-	73	107.0	73
COP-1:1	19.5	21.6	24.0	4.63	55	43	72	54	72.3	48
COP-1:2	19.5	21.5	24.0	4.64	53	45	82	41	74.7	49

^a^ Calculated using Equation (4) for (200) peaks, ^b^ Calculated from XRD data using Equation (5), ^c^ melting temperature belonging to PCL (I) and PTCL (II) part ^d^ ε-caprolactone content of the polymer, ^e^ Crystallinity calculated according to Equation (3).

**Table 2:** Main Thermogravimetric analysis (TGA) results of homo- and copolymers of CL and TCL.

Sample	T_max_^a^ (°C)			T_10%_^b^	Activation Energy (EA) ^c^, (kJ/mol)		
	I	II	III	(°C)	I	II	III
PCL	(212) ^d^	294		229	109	36	234
PTCL		262		224	99	142	60
COP-2:1	212	298	(328) ^e^	229	98	42	164
COP-1:1	224	303		206	93	43	116
COP-1:2	228	293		205	134	54	118

^a^ Maximum weight loss temperature determined at peaks of derivative weight loss (DTG) curves, ^b^ Temperature at which 10% weight loss occurred, ^c^ Estimated according to Broido method in corresponding ranges, ^d^ Observed as a peak with low intensity, ^e^ Observed as a shoulder.

**Table 3:** Yield and composition of chitosan-graft-polyester samples.

Sample	Yield ^a^	PCL Content ^b^	Chitosan Content
	(%)	(%)	(%, by weight)
CG-PCL	70	100	26
CG-PTCL	81	0	22
CG-B-2:1	54	28	34
CG-B-1:1	66	28	28
CG-B-1:2	64	15	28
CG-P-2:1	71	74	26
CG-P-1:1	64	49	29
CG-P-1:2	76	39	24

^a^ Calculated using Equation (6), ^b^ Estimated using FTIR.

**Table 4:** Main XRD and DSC results of chitosan-grafted homo- and copolymers.

Sample	2θ (110)		2θ (200)	Chain Distance ^a^	Χ_XRD_^b^	T_m, I_	T_m, IIa_	CL ^c^	ΔH_m_	Χ_DSC_^d^
	(°)		(°)	(Å)	(%)	(°C)	(°C)	(%, mol/mol)	(J/g)	(%)
CG-PCL		21.5	23.8	4.67	57	57	-	100	38.2	37
CG-PTCL	19.6		24.2	4.60	33	-	97	0	32.5	26
CG-B-2:1	19.5	21.5	24.0	4.64	29	56	97	28	10.6	10
CG-B-1:1	19.5	21.6	24.0	4.63	37	56	97 (sh)	28	51.7	46
CG-B-1:2	19.6		24.0	4.63	32	58	97	15	65.4	57
CG-P-2:1		21.4	23.8	4.68	28	53	-	74	21.9	20
CG-P-1:1	19.7	20.9	24.1	4.62	35	26 (br)	81	49	12.0	11
CG-P-1:2	19.6		24.1	4.62	20	26 (br)	82	39	35.2	30

^a^ Calculated using Equation (4) for (200) peaks, ^b^ Calculated from XRD data using Equation (5), ^c^ ε-caprolactone content of the polymer, ^d^ Crystallinity calculated according to Equation (3).

**Table 5:** Main TGA results of chitosan-grafted homo- and copolymers.

Sample	T_max_^a^ (°C)				T_10%_^b^	Activation Energy (EA) ^c^, (kJ/mol)			
	I	II	III	IV	(°C)	I	II	III	IV
CG-PCL		288 ^d^	310	417	262	106	134	14	55
CG-PTCL		266, 286 ^d^		410	225	122	-	12	57
CG-B-2:1		238, 289	(317) ^d^	416	227	131	65	16	56
CG-B-1:1		255, 291	324	414	228	129	56	17	60
CG-B-1:2		258, 288		412	227	127	50	14	57
CG-P-2:1	225	261, 290		411	243	135	107	11	55
CG-P-1:1	231	289		410	221	150	70	13	59
CG-P-1:2	225	282		406	212	158	52	15	54

^a^ Maximum weight loss temperature determined at peaks of DTG curves, ^b^ Temperature at which 10% weight loss occurred, ^c^ Estimated according to Broido method in corresponding ranges, ^d^ Observed as a shoulder.

## Abbreviations

FTIR	Fourier transform infrared
XRD	X-Ray diffraction
NMR	Nuclear magnetic resonance
DSC	Differential scanning calorimetry
TGA	Thermogravimetric analysis
DTG	Derivative weight loss
SEC	Size exclusion chromatography
M_n_	Number average molar mass
Đ	Dispersity
T_m_	Melting temperature
T_max_	Maximum degradation temperature
T_x%_	Temperature at which x% weight loss occurred
E_A_	Estimated activation energy
PCA	Principal component analysis
χ_DSC_	Crystallinity based on DSC measurements
χ_XRD_	Crystallinity based on XRD measurements
CL	ε-Caprolactone
TCL	ε-Thiocaprolactone
PCL	Poly(ε-caprolactone)
PTCL	Poly(ε-thiocaprolactone)
HDI	Hexamethylene diisocyanate
TBD	1,5,7-triazabicyclo[4.4.0]dec-5-ene
ROP	Ring opening polymerization
COP-x:y	Poly(ε-caprolactone-co-ε-thiocaprolactone) copolymer with comonomer feed ratio of x CL: y TCL
CG-PCL	Chitosan-graft-poly(ε-caprolactone) copolymer
CG-PTCL	Chitosan-graft-poly(ε-thiocaprolactone) copolymer
CG-B-x:y	Chitosan-graft-x poly(ε-caprolactone):y poly(ε-thiocaprolactone) with blending method
CG-P-x:y	Chitosan-graft-x poly(ε-caprolactone-co-poly(ε-thiocaprolactone) with COP-x:y copolymers
